# The Diagnosis of Syphilitic Skin Affections

**Published:** 1898-02-05

**Authors:** C. F. Marshall

**Affiliations:** Resident Medical Officer, London Lock Hospital


### Medical Progress and Hospital Clinics.

[*The Editor will be glad to receive offers of co-operation and contributions from members of the profession. All letters should be addressed to* The Editor, at the Office, 28 & 29, Southampton Street, Strand, London, W.C.]

### THE DIAGNOSIS OF SYPHILITIC SKIN AFFECTIONS.

By C. F. Marshall, M.D., F.R.C.S., Resident Medical Officer, London Lock Hospital.

(*Continued from page* 309.)

**Bullous Eruptions or Pemphigus.**— Pure syphilitic pemphigus is almost confined to cases of congenital syphilis, being almost unknown in adults. When it occurs it chiefly affects the palms of the hands and the soles of the feet. It is true that rupia may begin as a purulent bullous eruption, but the characteristic appearance of rupia is soon developed, and such cases cannot be confounded with a true pemphigus.

**Acneform Eruptions.**—Acne of syphilitic origin is rare and usually only occurs mixed with other eruptions. When present it differs from ordinary acne in the absence of comedones, and the darker colour.

**Pigmentary Eruptions.**—Apart from the pigmentary stains left by the remains of former secondary rashes, there is apparently a distinct pigmentary syphilide, which we may call leuco-melanoderma. This is more common in women, and generally affects the back of the neck. It consists of irregular spots, discrete or confluent, of a dark brown colour, the skin between being of an abnormally white colour.

**Alopecia.**—This, according to Crocker, may occur in four ways: (*a*) A secondary general thinning of the hair from malnutrition. (*b*) A patchy alopecia resembling alopecia areata. (*c*) A circumscribed eruption from the implication of hair follicles on the site of former eruptions. (*d*) Tertiary ulceration, destroying the whole thickness of the skin.

**Affection of the Nails.**—In syphilis, onychia and perionychia sometimes occur. The former is due to defect in nutrition; the latter to extension of a squamous lesion to the matrix, or to gummatous infiltration.

**Purpura.**—Secondary skin rashes in syphilis occasionally become purpuric, but this is rare, and usually due to constitutional disturbance apart from syphilis.

**Tubercular Lesions, or Cutaneous Gummata.**—These are convex projections of the skin usually occurring in tertiary syphilis, but they may develop in the secondary stage. They often ulcerate and coalesce, forming the so-called "syphilitic lupus." Diagnosis: This has to be made from lupus. The latter differs in having much slower growth, in being usually present in childhood or adolescence, and in the presence of the characteristic "apple-jelly" deposit of tubercle round the edges of the ulcers.

**Subcutaneous Gummata.**—These are common in late secondary and early tertiary syphilis, especially on the front of the legs over the tibia. They have to be diagnosed from rheumatic nodules; this is usually easy, as they seldom occur without other signs of syphilis.

**Syphilitic Ulceration.**—This may follow (1) cutaneous pustules; (2) cutaneous and subcutaneous gummata; (3) rupia. The ulcers are usually circular, with well-defined, sharply-cut edges, and a greyish floor. They often become uniform by extension on one side, while cicatrisation occurs on the opposite side. Confluence of several ulcers causes the well-known serpiginous ulceration. Gummatous ulcers form deep excavations, with crater-like walls. All syphilitic ulcers are characterised by the formation of greenish crusts. Rupia commences as a purulent bulla, which soon ulcerates and becomes covered with a scab. The ulceration extends, and fresh scabs form underneath, resulting in a conical series of scabs one over the other, resembling a limpet shell. The diagnosis of syphilitic ulcerations is usually easy by attention to the above points.

The distinction from lupus has been already mentioned. Gummata differ from tubercular nodules by the greater amount of fibrous tissue in the former, and the greater tendency to caseation of the broken down inflammatory products of the latter. Tubercular ulcerations are much more irregular, and have usually thin ragged edges.

**Condylomata.**—These form one of the typical lesions of syphilis. They are moist, flat papules situated on parts where the skin is moist, especially about the external genitals and anus. They are simply a development from macular or papular spots due to the moisture of the part. They can scarcely be mistaken for anything else. Gonorrhœal warts and papillomata are more pedunculated and papilliform. Sometimes gonorrhœal warts develop on the top of condylomata, forming the so-called warty condyloma.

